# Factors affecting residency rank-listing: A Maxdiff survey of graduating Canadian medical students

**DOI:** 10.1186/1472-6920-11-61

**Published:** 2011-08-25

**Authors:** Tao Wang, Benson Wong, Alexander Huang, Prateek Khatri, Carly Ng, Melissa Forgie, Joel H Lanphear, Peter J O'Neill

**Affiliations:** 1 Department of Laboratory Medicine and Pathobiology, University of Toronto, 1 King's College Circle, Floor 5, Toronto, Ontario, Canada; 2 Department of Medicine, University of Ottawa, 451 Smyth Rd, Ottawa, Ontario, Canada; 3 Department of Anesthesia, University of Toronto, Room 121, Fitzgerald Building 150 College Street, Toronto, Ontario, Canada; 4 Department of Medicine, University of Toronto, Suite RFE 3-805, 200 Elizabeth St., Toronto, Ontario, Canada; 5 Department of Family and Community Medicine, 500 University Avenue, Floor 5, Toronto, Ontario, Canada; 6 Department of Medical Education, Northern Ontario School of Medicine, 955 Oliver Road, Thunder Bay, Ontario, Canada; 7 Department of Obstetrics and Gynecology, 68 Barrie Street, Queen's University, Kingston, Ontario, Canada

## Abstract

**Background:**

In Canada, graduating medical students consider many factors, including geographic, social, and academic, when ranking residency programs through the Canadian Residency Matching Service (CaRMS). The relative significance of these factors is poorly studied in Canada. It is also unknown how students differentiate between their top program choices. This survey study addresses the influence of various factors on applicant decision making.

**Methods:**

Graduating medical students from all six Ontario medical schools were invited to participate in an online survey available for three weeks prior to the CaRMS match day in 2010. Max-Diff discrete choice scaling, multiple choice, and drop-list style questions were employed. The Max-Diff data was analyzed using a scaled simple count method. Data for how students distinguish between top programs was analyzed as percentages. Comparisons were made between male and female applicants as well as between family medicine and specialist applicants; statistical significance was determined by the Mann-Whitney test.

**Results:**

In total, 339 of 819 (41.4%) eligible students responded. The variety of clinical experiences and resident morale were weighed heavily in choosing a residency program; whereas financial incentives and parental leave attitudes had low influence. Major reasons that applicants selected their first choice program over their second choice included the distance to relatives and desirability of the city. Both genders had similar priorities when selecting programs. Family medicine applicants rated the variety of clinical experiences more importantly; whereas specialty applicants emphasized academic factors more.

**Conclusions:**

Graduating medical students consider program characteristics such as the variety of clinical experiences and resident morale heavily in terms of overall priority. However, differentiation between their top two choice programs is often dependent on social/geographic factors. The results of this survey will contribute to a better understanding of the CaRMS decision making process for both junior medical students and residency program directors.

## Background

In Canada, the vast majority of graduating medical students secure their residency placement through the Canadian Residency Matching Service (CaRMS) [1]. After the students have undergone the application and subsequent interview process, a match is conducted using an algorithm that considers both student and program rank lists. Rank preferences of the former are given priority over the latter [1]. Through better understanding of this decision process, changes may be made to the benefit of both applicants and residency programs.

Many factors influence how students rank residency programs, including geographic, social, lifestyle, and academic factors [2-13]. However, the relative significance of these factors has not been well studied in Canada. Previous studies in this area were limited by small sample sizes and usually restricted to one specialty [3-8,11]. Furthermore, it is largely unknown how students specifically differentiate between their top two program choices. In 2009, approximately 63% of applicants matched to their top choice program while only 13% and 7.6% matched to their second and third choice programs respectively [1]. Therefore, the characteristics that distinguish top choice from second choice programs have the greatest absolute impact on where an applicant is most likely to match. An individual program's ability to modify these distinguishing factors will vary, and this issue has not been previously explored.

A few existing studies have examined potential gender influences but these have been limited to subspecialty populations [2,3,5]. We hypothesize that in a general applicant pool, there will be minimal significant differences between male and female applicant priorities. There are also no studies to our knowledge that have looked at differences between family medicine and specialty applicants. Given differences in eventual patient care roles, it is theorized that specialty residents will, as a group, place greater emphasis on academic factors such as research and academic reputation.

All previous studies on this topic employed surveys using Likert-style rating scales [2-14]. While prevalent in survey research due to ease of use, Likert style rating possess many systematic biases that adversely affect statistical analysis and overall validity. Respondents often cluster their answers and rarely utilize the full spectrum of the scale; these tendencies are known as level and dispersion bias respectively [15]. Level bias also contributes to a non-standardized distribution of "mean" answers; thus those who cluster their responses at the extreme ends of the scale will be disproportionately represented in the final numerical analysis. Lastly, the Likert scale is ordinal, meaning it only conveys order and not magnitude; however, in medical literature it is often analyzed using t-tests or ANOVA as though it is an interval scale [2,3,9,10]. This practice, while widespread, is statistically controversial [16-18].

This study employs the discrete-choice methodology known as "Maxdiff" or "best-worst" scaling to determine the relative importance of various factors. Maxdiff methodology is based on the theoretical framework of discrete choice mathematics developed by Nobel laureate Daniel McFadden [19-23]. This technique provides a solution to most of the problems of rating scale surveys through inherent standardization of mean responses and utilization of the full spectrum of the final numerical scale. It also forces respondents to make choices and compromises, which ultimately leads to better differentiation between factors. The main drawbacks to Maxdiff methodology are that surveys are more time consuming to construct and analyze than with traditional rating scales, but we felt its strengths justified its use.

The primary focus of our study is the overall relative importance of various factors students consider when deciding between residency programs. We will also investigate how students distinguish between their top two program choices, as well as the influence of demographics and career choices on applicant preferences. The goal of our study is to provide those involved in the post-graduate education process with a better understanding of applicant priorities. We hope to identify areas where residency programs may make improvements so that they can be more appealing to applicants.

## Methods

Our study population included graduating medical students from all six Ontario medical schools who were participating in the CaRMS 2010 match cycle. Ontario was chosen since it is the province with the most number of medical schools and students. We also hoped students would perceive a provincial level study to be more relevant and thus be more eager to participate. Approval for the study was obtained from the Institutional Human Research Ethics Boards associated with the six schools (University of Ottawa, University of Toronto, University of Western Ontario, Queen's University, McMaster University, and Northern Ontario School of Medicine).

### Survey Administration

Beginning three weeks prior to the CaRMS match date (March 8, 2010), graduating medical students from all six Ontario medical schools were invited by email to participate in the study. At that time, students were in the process of ranking their program choices. Each student was provided an anonymous and unique response code for completing an online survey administered through the services of Survey Gizmo (Boulder, CO, USA). Informed consent was obtained electronically. An incentive was provided in the form of a single randomly drawn $200 cash prize. Completion of the survey was not necessary for an invitee to be eligible for the draw. One week prior to the survey completion date, a single set of reminder emails was sent to all participants to increase response rates. The survey ended on March 8, 2010, prior to the release of CaRMS results so that the match results would not bias responses.

### Survey Design

Basic demographic data was collected, including questions about age, gender, marital status and specialty choice. The bulk of our survey focused on 13 factors selected based on a literature review. We chose factors that were commonly cited as influential and that had minimal co-variance with other factors. Some studies have used terms such as "fit for program" or "geographic location" which are obviously rated highly but can be ambiguous in meaning [4,5,10]. We attempted to decrease ambiguity by avoiding generic terms such as "geographic location"; instead, we separated the factor into social aspects (friends/family) and city characteristics. We also excluded factors that were previously found to be unimportant, such as benefits package, moonlighting opportunities, and amount of interaction with medical students [10].

**Figure 1:**
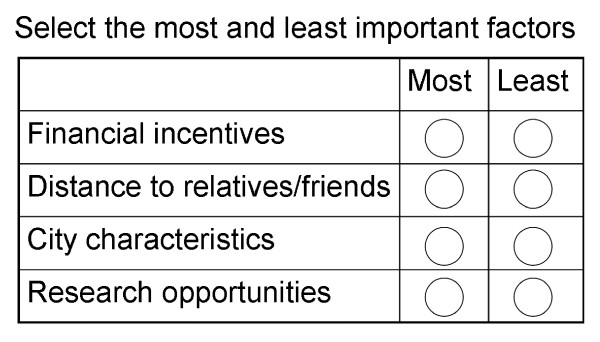
**A typical MaxDiff style question as seen on the survey**. Respondents choose one factor per set of four as the most important and one as the least important.

These factors formed the basis of 13 questions styled in a Maxdiff format (Figure 1) [19-23]. Each question contained a subset of 4 factors; the respondent would choose the most and least important factors within the subset. The question sets were balanced in factor frequency, positional frequency and orthogonality. This means that each factor appeared 4 times in total, and was paired with any other given factor once. This ensures that all factors have the chance to be compared against any other specific factor at least once within the same set. This arrangement was achieved by building a question matrix using the balanced incomplete block design technique described elsewhere [14,24].

The survey also inquired about the principle reason why respondents did not choose their second-ranked program, using drop-list style questioning. The same 13 factors were built into the drop-list but stated in a negative sense. For example, the second ranked program was not chosen because "it had less variety of clinical experiences".

The survey was piloted with a subset of six volunteer final year medical students from a single school. Their suggestions helped us revise instructions and better clarify potentially confusing aspects of the survey. The scientific methodology and content was not significantly changed.

### Statistical Analysis

Only respondents who completed the demographics and Maxdiff sections in full were included in the analysis. Maxdiff data was analyzed using a scaled simple count method, which is easy to comprehend and has been previously validated [19,21]. Each time a factor was chosen as "most important", its count was incremented by one. Conversely, when a factor was chosen as "least important", its count was decremented by one. This allowed each respondent to generate a "score" for each factor. Since each factor appears in exactly four subsets, the scores ranged from +4 to -4. These scores were averaged over the sample to obtain mean scores for each factor, demonstrating their relative importance overall. To examine subgroups, we separated the sample between males and females, as well as between family medicine applicants versus specialty applicants. Given the ordinal nature of the scale, statistical significance was determined using the Mann-Whitney test. P-values ≤0.05 corresponded to statistical significance. Analysis was performed using the software PAST (Oslo, Norway).

Data was also analyzed to elucidate the top reasons why applicants did not choose their second ranked program. As an individual respondent could only choose one answer from the drop-list menu, the results were organized as percentages. The factors were subcategorized as "modifiable", "potentially modifiable", and "non-modifiable" based on the potential control a program may exert over that factor.

## Results

The survey produced full responses from 339 of 819 eligible Ontario graduating medical students for an overall response rate of 41.4%. The respondents' characteristics are summarized in Table 1 and appear to be similar to the available reported values for all CaRMS applicants [1].

**Table 1:** Respondent characteristics in comparison to total CaRMS pool

	No. of Respondents (%)	CaRMS applicants (%)
**Total**	339	2438
**Age**		
≤24	54 (16)	
25-26	127 (39)	
≥27	148 (45)	
**Gender**		
Male	127 (39)	1019 (42)
Female	202 (61)	1419 (58)
**Marital status**		
Single	233 (71)	
Married/Common-law	96 (29)	
**Career choice**		
Family medicine	121 (37)	775 (32)
Specialty	207 (63)	1663 (68)

The Maxdiff section of the survey was scaled for each respondent using the simple count method. Table 2 displays the mean scaled score of all the factors in the survey. Overall, the variety of clinical experiences, resident morale, and the distance to relatives were given the heaviest emphasis by applicants. In contrast, factors such as research opportunities, finance, and parental leave attitudes were considered least important.

**Table 2:** Overall scaled Maxdiff survey scores for various factors

Factor (Short form)	Mean Maxdiff Score (± Standard error)
Variety of clinical experiences (Variety)	1.85 (0.086)
Resident morale (Morale)	1.66 (0.081)
Distance to relatives (Relatives)	1.49 (0.111)
City characteristics (City)	0.87 (0.111)
Academic reputation (Academic)	0.71 (0.104)
Spouse/partner's preferences (Spouse)	0.38 (0.160)
Quality of faculty (Faculty)	0.30 (0.092)
Interview experience (Interview)	-0.40 (0.098)
Intensity of work schedule (Schedule)	-0.44 (0.080)
Hospital facilities (Hospital)	-0.45 (0.081)
Research opportunities (Research)	-1.37 (0.106)
Financial incentives (Finance)	-2.18 (0.076)
Parental leave attitudes (Parental)	-2.42 (0.087)

Differences between male and female applicants were examined (Table 3). Some statistically significant differences were found, namely regarding academic reputation, research opportunities and parental leave attitudes. However, none of these factors were rated highly by either group.

**Table 3:** Comparison of Maxdiff scaled scores by gender and by specialty choice

Factor	Mean Maxdiff Score (±SE)					
	
	Gender			Specialty Choice		
	Male	Female	p-score†	Family Medicine	Specialties	p-score†
Variety	1.80 (0.140)	1.89 (0.108)	0.545	2.40 (0.129)	1.53 (0.106)	<0.001*
Morale	1.54 (0.136)	1.74 (0.100)	0.204	1.40 (0.126)	1.82 (0.104)	0.019*
Relatives	1.18 (0.192)	1.68 (0.133)	0.053	1.48 (0.180)	1.51 (0.140)	0.788
City	0.84 (0.164)	0.89 (0.148)	0.695	0.81 (0.183)	0.89 (0.139)	0.801
Academic	1.06 (0.168)	0.48 (0.130)	0.002*	0.17 (0.158)	1.04 (0.131)	<0.001*
Spouse	0.61 (0.245)	0.23 (0.209)	0.231	0.90 (0.275)	0.07 (0.194)	0.015*
Faculty	0.43 (0.157)	0.22 (0.113)	0.213	-0.10 (0.135)	0.53 (0.121)	<0.001*
Interview	-0.46 (0.166)	-0.36 (0.120)	0.526	0.13 (0.149)	-0.71 (0.124)	<0.001*
Schedule	-0.62 (0.118)	-0.33 (0.106)	0.190	-0.31 (0.127)	-0.53 (0.102)	0.159
Hospital	-0.35 (0.128)	-0.51 (0.106)	0.301	-0.26 (0.131)	-0.57 (0.103)	0.054
Research	-1.01 (0.183)	-1.60 (0.127)	0.012*	-2.38 (0.140)	-0.77 (0.131)	<0.001*
Financial	-2.27 (0.117)	-2.12 (0.099)	0.345	-2.06 (0.119)	-2.25 (0.098)	0.113
Parental	-2.76 (0.114)	-2.20 (0.120)	0.006*	-2.17 (0.151)	-2.57 (0.105)	0.028*

† p-scores were obtained using the Mann-Whitney test. * indicates significant differences with p-score ≤0.05

Applicants choosing family medicine were compared with other specialties (Table 3). Numerous statistically significant differences were found. Family medicine applicants appeared to rate variety of clinical experiences higher and resident morale lower in importance by comparison. Specialty applicants were more concerned with academic reputation, the quality of the faculty, and research opportunities.

Respondents were asked about the main reason why they didn't choose their second choice program over their first. We called this the "distinguishing factor". The results displayed differences from overall importance ratings. The top reasons why applicants did not choose their second ranked program first were greater distance to relatives, less desirable city, and less preferred by spouse/partner. It is interesting to note, as shown in Table 4, that at least 63.6% of the time, the reason was entirely outside the control of the program. Only 12.7% of the chosen reasons can be classified as a modifiable factor.

**Table 4:** Top reasons for why the respondents' 2^nd ^ranked program was not their 1^st ^choice

Distinguishing Factor	Top reason second choice program was not first choice (%)
Non-modifiable*	
Greater distance to relatives	23.33
Less desirable city	20.00
Less preferred by spouse/partner	19.67
Fewer financial incentives	0.67
	
**Potentially modifiable**	
Less variety of clinical experiences	9.33
Less academically reputable	6.33
Less impressive faculty	1.00
Less impressive hospital facilities	0.33
	
**Modifiable**	
Less desirable work schedule	5.00
Poorer resident morale	4.33
Less impressive interview experience	2.33
Fewer research opportunities	1.00
Less suitable parental leave attitudes	0
	
**Other**	6.67

*Non-modifiable factors are issues over which the program has no control. Potentially modifiable factors constitute issues where the program would have difficulty to modify. Modifiable factors represent issues that the program can have direct control over.

## Discussion

Our survey has demonstrated that applicants highly value "variety of clinical experiences" but nonetheless often distinguish between top programs based on social/geographic factors. There were no major differences based on gender, but there were several differences between family medicine and specialty applicant priorities. Most of these results are novel in this area of research as well as in this population and shed some light on CaRMS applicant decision making.

From the results of the Maxdiff section (Table 2), the top three factors overall were the variety of clinical experiences, resident morale, and distance to relatives. Conversely, most lifestyle factors such as financial incentives, work schedule, and parental leave were not considered important. It would seem that applicants favour program quality and social factors over lifestyle factors. These general trends are consistent with other studies [3,6,10,12].

There were very few differences between male and female applicants, except that males tended to put greater emphasis on academic reputation and research (Table 3). While females placed more emphasis on parental leave attitudes, both groups ranked that factor last. Thus, overall, male and female medical students have similar priorities when selecting a residency program, which is consistent with other studies [2,3].

Family medicine and specialty applicants do display several statistically significant differences (Table 3). Family physicians certainly require a broad range of knowledge and thus it is not surprising that family medicine applicants put greater emphasis on the variety of clinical experiences. The greater emphasis by specialty applicants on resident morale may reflect the fact that they spend considerably more time on-service and usually have longer residency programs. There is a disproportionate ratio of specialists to family physicians with appointments at academic centers as staff physicians [25]. Our results were consistent with this work environment preference, as specialty residents on average placed greater emphasis on academic factors such as quality of faculty, research opportunities and academic reputation. Specialty applicants also gave the interview experience a lower priority. It is plausible that applicants to more competitive specialties may have felt content to get into their chosen specialty regardless of location or program; thus leading to less emphasis on the interview experience.

Table 4 displays respondent selections of the principal reason why they didn't choose their second ranked program, also known as the distinguishing factor. Given that most people match to their first choice program, the choice to rank a program second causes the greatest statistical drop in match probability. It is interesting to note that the frequency of the distinguishing factors has a different order than their overall priorities. For instance, while variety of clinical experiences was ranked first overall, it was the distinguishing factor only 9.33% of the time. Most applicants chose factors that were entirely outside the control of the program, such as social and geographical factors. This discrepancy suggests that while respondents highly valued factors such as variety of experiences and positive resident morale, many programs fulfilled their expectations in these areas. Thus, when it came down to a decision between their top choices, most chose the program that better suited their social and geographical situations. It may seem discouraging to program directors that they appear to have limited control over this final decision. Nonetheless, the high overall values placed on many controllable factors indicate that programs need to meet those criteria to be seriously considered. Some studies on resident burnout, work hours, and morale have found positive benefits from options such as hiring physician assistants and the limitation of resident work hours [26,27]. In Canada, not all provincial regulatory bodies have set maximum duty hours and programs do have some influence in the work schedules and hours of their residents. Clinical variety is a more difficult factor to modify, as some centers are simply limited by the patient volumes they see. However, innovations such as use of simulations and the creation of dedicated "medical procedure rotations", have been demonstrated to increase resident confidence in scenarios they otherwise seldom encounter [28,29].

There are limitations to our study that affect the way the results may be interpreted. Our sample was drawn from the most populous province in Canada; thus geographic factors may become more of an issue when considering a national sample. Quantitative and qualitative differences in the application process, post-graduate training programs, and the health care system make international comparisons difficult. For instance, students in the United States would have a greater number of residency programs to choose from, including programs outside of their national matching service. This can certainly affect the factors that influence their decisions. Our response rate of 41% is another limitation, but this is comparable to other published student surveys on this topic of similar scale [2-5,9-11]. Also, the demographic similarity of our sample to that of the CaRMS applicant pool indicates that the survey respondents were representative of the population of interest (Table 1). Lastly, our survey focused on 13 factors, while certainly other issues may influence applicants. However, as previously mentioned we chose a range of factors found to be of potential importance by previous literature and excluded those previously found to be of very low priority. We aimed to create a comprehensive questionnaire while at the same time avoiding low yield questions that may increase participant fatigue and drop-out.

The strengths of our study include its novel methodology as well as several unique findings. Maxdiff methodology leads to a standardized numerical scale with results that disperse across the full spectrum of the scale. This eliminates the systematic level and dispersion biases that affect the Likert style rating scales used in almost all other studies [14]. Maxdiff also forces respondents to make decisions between a set of factors, thus providing better differentiation between factors when compared to rating scales [10,14]. To our knowledge, this is the only survey to date on this topic which asks how applicants distinguish between their top programs. Moreover, this study provides unique insights into the different values of family medicine and specialty applicants.

## Conclusions

This survey has contributed to a better understanding of which aspects of the selection process are emphasized by graduating medical students. Residency program quality issues, such as variety of clinical experiences and resident morale, are important considerations, but social and geographic characteristics tend to separate the top choice from the second choice. Male and female applicants have similar priorities in program selection. While family medicine applicants especially value clinical variety, specialty applicants emphasize academic factors such as research opportunities and program reputation. Lifestyle factors such as financial incentives or work schedule have little impact on applicants. Residency programs will hopefully make efforts to improve on the modifiable characteristics in order to better appeal to applicants.

## Competing interests

The authors declare that they have no competing interests.

## Authors' contributions

TW conceived the study and was involved in study design, implementation, analysis and manuscript editing. BW and PJO contributed to the design of the study protocol. AH, PK, CN, MF, and JHL contributed to study implementation and analysis/interpretation of data. BW, AH, PK, and CN drafted the manuscript. All authors critically reviewed the manuscript regarding its intellectual content and approved the final version submitted for publication.

## Pre-publication history

The pre-publication history for this paper can be accessed here:

http://www.biomedcentral.com/1472-6920/11/61/prepub
